# Supplementary material for: Engineered osteoclasts as living treatment materials for heterotopic ossification therapy
Source: Nat Commun. 2021 Nov 3;12:6327. doi: 10.1038/s41467-021-26593-1 (PMC8566554; doi:10.1038/s41467-021-26593-1)
Supplement: Supplementary file 1 — Supplementary Information [file 41467_2021_26593_MOESM1_ESM.pdf]

Supporting Information

Engineered Osteoclasts as Living Treatment Materials for Heterotopic Ossification Therapy

Wenjing Jin[1,2], Xianfeng Lin[3,4], Haihua Pan[5], Chenchen Zhao[3,4], Pengcheng Qiu[3,4], Ruibo Zhao[6], Zihe Hu[2], Yanyan Zhou[2], Haiyan Wu[2], Xiao Chen[7,8], Hongwei Ouyang[7,8], Zhijian Xie[2], Ruikang Tang[1,5]**

1.Experimental Section

Tartrate-resistant acid phosphatase (TRAP) staining. TRAP staining (Sigma) was used to evaluate OCs differentiation. BMM cells were seeded onto 96-well plates (Corning) at a density of 1×10^5 cells/ml and cultured in α-MEM (Gibco, USA) supplemented with a 25 μg/l M-CSF and 50 μg/l RANKL as indicated in the results section for 6 days. OCs were digested with 0.25% EDTA-trypsin (Gibco, USA) for 3-5 min and then seeded onto 96-well plates (Corning) in α-MEM supplemented with a 25 μg/l M-CSF and 50 μg/l RANKL for 24 h. Cells were fixed with 4% formaldehyde for at least 15 min at room temperature and stained for TRAP.

Supplementary Table1: TRAP staining solution (in 50-ml beakers)

Reagent	Volume/ml
Fast Garnet GBC Base Solution	0.5
Sodium Nitrite Solution	0.5
Deionized water prewarmed to 37 °C	45

Naphthol AS-BI Phosphate Solution	0.5
Acetate Solution	2
Tartrate Solution	1

Multinucleated TRAP-positive cells with at least 3 nuclei were scored as OCs.

CCK8 assays (cytotoxicity of TC). OCs were plated in 96-well plates at 1×10^4 cells/well. At 24 h, different TC concentrations (10 μg/ml; 20 μg/ml; 40 μg/ml; 80 μg/ml; 160 μg/ml; 320 μg/ml; 640 μg/ml) were applied. Then, cells were cultured in α-MEM (10% FBS) at 37 °C in a humidified incubator with 5% CO_2 for an additional 24 h. Subsequently, 20 μl of CCK8 reagent (Beyotime, China) was added, and the cells were incubated at 37 °C for an additional 1-2 h. The absorbance of the plates was read at 450 nm with a microplate reader (BioTek, USA).

Scanning electron microscopy (SEM). The sample was first fixed with 2.5 % glutaraldehyde in phosphate buffer (1×PBS, 10 mM, pH 7.2) for more than 4 h, washed three times with phosphate buffer for 15 min each time, post-fixed with 1 % OsO_4 in phosphate buffer for 1-2 h and washed three times with PBS for 15 min each time. The sample was first dehydrated by a graded series of ethanol (30 %, 50 %, 70 %, 80 %, 90 % and 95 %) for approximately 15 min each time and then dehydrated by alcohol twice for 20 min each time or stored in alcohol. The sample was dehydrated in a Hitachi Model HCP-2 critical point dryer. The dehydrated sample was coated with gold-palladium in a Hitachi Model E-1010 ion sputter for 4-5 min and observed on a Hitachi Model SU-8010 SEM.

Sample processing procedures for transmission electron microscopy (TEM). The sample was prepared according to the SEM processing procedures mentioned above. Then, skeletal samples were decalcified in 14.3 % $EDTA_2Na$ (pH=7.4, containing 25 g of $EDTA_2Na$, 2.5 g of NaOH, 175 ml of ddH_2O) for 1 week. The specimen was placed in a 1:1 mixture of absolute acetone and the final Spurr resin mixture for 1 h at room temperature and then transferred to a 1:3 mixture of absolute acetone and the final resin mixture for 3 h to obtain a final Spur resin mixture overnight. The specimen was placed in an Eppendorf tube containing the Spurr resin and heated at 70 °C for more than 9 h. The specimen was sectioned using a LEICA EM UC7 ultratome and stained with uranyl acetate and alkaline lead citrate for 5 to 10 min before being observed with a Hitachi Model H-7650 TEM.

AIEgen probe pH sensing. An AIE™ pH (10 mM) stock solution was prepared with 10 μM AIE™ pH in 1 ml of DMSO. The stock solution was diluted 1000-fold. OCs or TC-OCs (2×10^4 per well) were seeded on one side of a well in a 48-well plate, and ectopic calcified tissue was seeded on the other side of the well. After culturing the OCs for one night, they were stained with the working concentration of 10 μM AIE™ pH for 2 h, and then the live cells were washed with PBS three times subjected to confocal laser scanning microscopy (CLSM). The following confocal imaging conditions are recommended: channel 1: excitation/emission = 405/440 - 525 nm; channel 2: excitation/emission = 488/561 - 659 nm. The nucleus was stained with Hoechst 33324 (Beyotime, China). After 4 days, the calcified tissue was collected and fixed in 4 % paraformaldehyde for 15 min for SEM observation.

TC molecules on cell surface. The number of TC molecules attached to the TC-OC cell membrane was quantified at working concentrations (160 μg/ml) by Varioskan Flash (Thermo) based on their autofluorescence ability. TC-OCs were prepared (100 μl, 2×10^5 cells/ml) and resuspended in a 0.9% NaCl solution before being added to 96-well plates. The concentration of TC (the value is 20 μg/ml) on cell surface was obtained according to OD data (the value is 0.2) of solution measured by Microplate reader. The number of TC molecules on the cell membrane was calculated by using a standard linear calibration curve of TC solution (100 μl; 10 μg/ml, 20 μg/ml, 40 μg/ml, 80 μg/ml and 160 μg/ml).

The results showed that each cell was modified with approximately 1.35×10^{11} TC molecules form equation (1), (2) and (3).

C_{TC}=20 μg/ml; V=100 μl

$$m_{TC}=C\times V=20\ \mu g/ml \times 0.1\ ml = 2\ \mu g \quad (1)$$

$$N_{total}=n \times N_A=\frac{N_A\times m_{TC}}{M_{TC}}= 6.02\times10^{23} \times 2 \times 10^{-6}/444.5 \approx 2.7 \times 10^{15}\ \text{molecules} \quad (2)$$

$$N_{tc/cell}= \frac{N_{total}}{N_{cell}} =2.7 \times 10^{15}/2 \times10^{4}=1.35 \times 10^{11}\ \text{molecules} \quad (3)$$

C_{TC} is the concentration of TC on the OC cell membrane surface; V is the volume of cell suspension; N_{total} is the total number of tetracycline molecules; m_{TC} represents the mass of the tetracycline molecule; M_{TC} represents the mass of the tetracycline molecule relative molecular mass; N_A *is the Avogadro's number*; N_{cell} is number of OCs and $N_{tc/cell}$ is number of tetracycline molecules on every OC.

Animal model. All animals were maintained at the Animal Facility of the Department of Orthopaedic Surgery, Sir Run Run Shaw Hospital, Zhejiang University School of Medicine. The experimental protocols were reviewed and approved by the Department of Orthopaedic Surgery, Sir Run Run Shaw Hospital, Zhejiang University School of Medicine (Number: SRRSH2021401 and 201801224). All animals were housed under a 12 h light/dark cycle at controlled room temperature of 22-24 °C and a relative humidity of 40-70 %. Briefly, 15 eight-week-old male Sprague–Dawley rats (body weight 290–330 g) were randomly assigned into three groups: blank (0.9% NaCl), OCs and TC-OCs. Then, tenotomy-induced HO was conducted [1]. All rats were deprived of food for 6 h before anaesthetization with 50 mg/kg pentobarbital sodium via intraperitoneal injection, after which they were fixed in the prone position. Under aseptic conditions, the left and right lower legs were arranged in the posterolateral position. The Achilles tenotomy was completely transected at the midpoint of the Achilles tendon. Both ends of the broken Achilles tendon were clamped repeatedly with vascular forceps 10 times, causing a certain amount of trauma, and the skin incision was sutured. After routine feeding and observation for 8 weeks, the animals were subjected to X-ray analysis (Faxitron MX-20, USA) and Micro-CT.

The standard intramuscular heterotopic ossification model refers to previous article.[2] Briefly, the clean parafilm was spread in a 10 cm cell culture dish, drop 150 μl collagen solution (Collgen I, Gibco) on it, and then spread 50 % ammonia in a desiccator for 4 hours to form a gel. Next step is to soak it in ionized water for about 30 min and change the water 3 times. Finally, make the pH neutral and freeze-dry it for 24 h to obtain a collagen gel, and 5 mm × 1 mm–thick collagen discs were prepared. A 5 μl containing 2 μg of recombinant human BMP-2 (GenScript) was adsorbed onto each disc. Longitudinal skin incisions were made on the medial surface of calf muscles in 36 6-week-old

SD rats. An intramuscular pocket was created microsurgically, and one BMP-2–collagen disc was placed in it. To induce heterotopic ossification, 0.3 μg per 10 μl cardiotoxin (Shanghai Boyao Biological Technology Co., Ltd. CAS: 9012-91-3) were injected into the anterior tibial muscles of rats; The skin was then sutured with 4-0 absorbable sutures. The subcutaneous model of muscle trauma–associated heterotopic ossification was obtained after 8 weeks. 12 eight-week-old males 8 the $Mkx^{-/-}$ transgenic mice were provided by Dr. Ronen Schweitzer (Oregon Health and Science University, Portland, OR).

Two-photon fluorescence microscopy analysis. To assess the location and shape of OCs/TC-OCs in vivo after injection, OCs/TC-OCs (100 μl, 10^6 cells/ml/500 g) were obtained and labelled with 5 μM Hoechst 33258 (blue, Beyotime, China) for 3 min, rinsed with PBS buffer, and resuspended in 0.9% NaCl. Calcified tissues were dissected after OCs/TC-OCs injected 0, 2 h. Frozen tendons from different groups were cryosectioned (100 μm) using Cryostar NX50 (Thermo Scientific). Then, samples were fixed in 4% paraformaldehyde for 30 min at room temperature. The sections were incubated with FOCM solution and gently shaken for 20 min at room temperature in 50 ml glass centrifuge tubes with 30 ml reagent.[3] After FOCM clearance, the samples were stained by Calcein (1 μg/ml) with were mounted and imaged on a confocal microscope (Olympus BX61). The location and shape of the ectopic calcifications and the density of OCs/TC-OCs in each Achilles tendon were clearly detected under two-photon excitation at 405 and 488 nm. The CLSM images were obtained by Imaris 9.5 (Batch) software.

Cell viability in vivo. Qualitative evaluations of OC and TC-engineered OC cell viability in vivo were performed by Bio-Real in vivo imaging (IVIS Spectrum, American). Achilles tenotomised rats (weight 500–600 g) were randomly divided into three groups (n=4) for analysis of cell viability in vivo. The blank group was treated with 0.9% NaCl (100 μl/500 g). OCs were encapsulated with TC via the surface engineering methods described above. OCs/TC-OCs were stained with Cell Trace Far Red DDAO-SE fluorescent tag (DDAO-SE, HH-C34553), a fixable, far-red-fluorescent tracer for long-term cell labelling, rinsed with saline solution three times, and then suspended in saline solution at a concentration of 10^6 cell/ml[4]. Briefly, 50 μg Cell-Trace was dissolved for 10 min in 25 μL dimethyl sulphoxide (DMSO). Subsequently, OCs/TC-OCs (10^6 cells/ml/500 g) were injected in situ to calcify the tendon and then subjected to Bio-Real in vivo imaging for 0 and 4 days.

Quantitative analysis of osteoclast and TC-engineered osteoclast cell viability in vivo were accomplished by flow cytometric method. OCs/TC-OCs (10^6 cells/ml/500 g) labeled with Hoechst 33258 (5 μM, Beyotime, China) were injected into the calcification in situ. In order to rinse off the injected OCs and engineered OCs, the injection site was marked. After 4 days, all rats were sacrificed by cervical dislocation. The marked site was transected completely and rinsed by 10 ml of normal saline for three times. The cell suspension was added to 5 ml Red Cell Lysis Buffer (C3702-120 ml, Beyotime Biotechnology) and lysed for 10 min, and then centrifuged at 2152 × *g* for 10 min. Finally, 300 μl of cell (10^5/ml) were labeled with LIVE/DEAD Viability/Cytotoxicity Kit (L3224, Invitrogen™) to subjected to flow cytometry. Calcein AM Excitation/emission wavelength: 494/517 nm, Standard filter set FITC.

Cranial and tibial bone resorption in vitro. To assess the capacity of TC-engineered cells for cranial and tibial bone resorption in vitro, the samples were subjected to micro-CT analysis at 60 kV and 133 μA (MiLabs, Netherlands). The cranial and tibial bones were obtained from SD rats (weight: 150-170 g, 6 weeks). The cranial bone was prepared at the same size (1×1 cm). The bone was randomly divided into three groups (n = 3) for the evaluation of bone resorption. The cranial bone was treated with 0.9% NaCl, OCs or TC-OCs. OCs or TC-OCs (2×10^4 per well) were seeded on one side of 48-well plate, and cranial bone was seeded on the other side of the well. Then, the cells were cultured in α-MEM (10% FBS) with 25 μg/l M-CSF and 50 μg/l RANKL. OCs or TC-OCs were seeded every two days for 2 weeks. The methods of tibia bone resorption were the same as above mentioned.

Cytokine response of bone formation and resorption. For serum biochemical analysis, approximately 2 ml of whole blood from each rat was harvested through the eyeball for serum analysis. Then, the blood samples were centrifuged at 5500 × g for 15 min after standing at room temperature for 3 h. Next, the serum was collected from the supernatant and examined for creatinine (CRE), urea nitrogen (BUN), aspartate transaminase (AST), alanine aminotransferase (ALT), alkaline phosphatase (ALP) and total calcium (Ca). The serum levels of BMP-2 (RA20063, Bioswamp), ALP (RA20082, Bioswamp), TNF-α (ab46070, Abcam) and IL-6 (ab21390, Abcam), TRAP (Cat. #JL21113) and CTX (Cat. #JL26864, Shanghai Jianglai Biological Technology Co., Ltd., China) were determined using an ELISA kit. TRAP, CTX, ALP and BMP-2 activity was determined in the supernatant of tendon tissue homogenate using ELISA kits (CTX ELISA Kit (Cat. #JL26864); TRAP ELISA Kit (Cat. #JL21113); BMP-2 ELISA Kit (Cat. #JL11722); (ALP ELISA

Kit, Cat. #JL26470); Shanghai Jianglai Biological Technology Co., Ltd., China). A TissuePrep homogenizer (Bio-Xplorer, TP-24 Tianjin, China) were used to homogenize 20 mg of tendon from different groups (blank; OCs TC-OCs) with 4 replicates. After centrifugation at 8609 × *g* for 10 min at 4 °C, the supernatant was collected for subsequent analyses. The absorbance value of the supernatant at 450 nm was measured with an microplate reader.

Statistical analysis. The statistical significance of all experiments was determined by Student's t-test and were presented as the mean ± standard deviation (SD) of three samples from independent analyses, as indicated. Statistical tests were performed by GraphPad Prism 8 for macOS (Version 8 (131)). P values were considered as statistically significant as follow: no significance (ns) $p > 0.05$, $*p < 0.05$, $**p < 0.01$, $***p < 0.001$, $****p < 0.0001$

2. Supplementary Figures

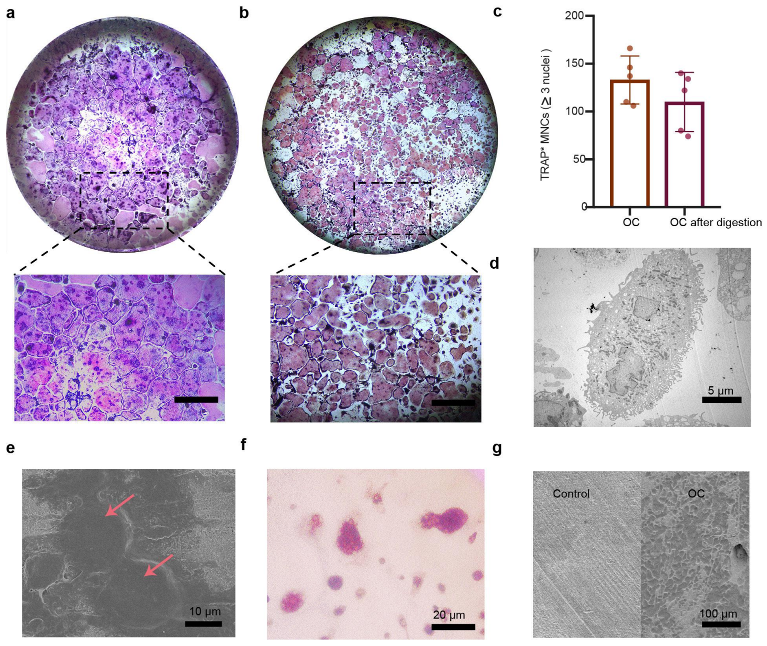

Supplementary Fig. 1. OCs differentiation and bone resorption capacity. (**a**) Representative TRAP staining images of OCs. Bar: 300 μm (**b**) TRAP staining of OCs after digestion. Bar: 300 μm (**c**) The number of mononuclear OCs (MNCs) was counted (n = 5). (**d**) Direct TEM images of untreated OCs; (**e**) SEM images of OCs generated in vitro on cortical bone slices from M-CSF and RANKL-treated BMMs; red arrows indicate OCs. (**f**) Representative TRAP staining images of OCs on bone pits. (**g**) Representative SEM images of resorption pits generated by OCs digested from mature OCs generated in vitro from M-CSF and RANKL-treated BMMs, and BMMs as control. Data are represented as mean ± SD. Source data are provided as a Source Data file.

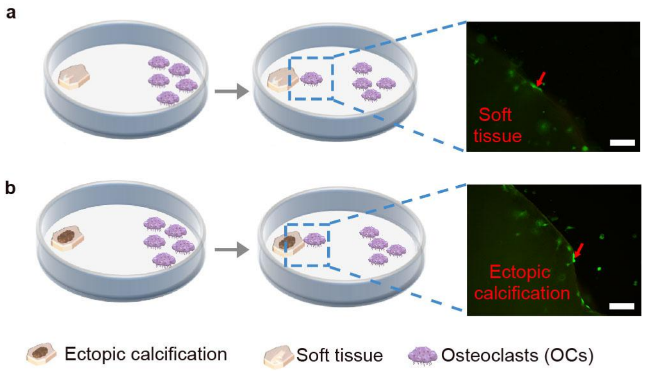

Supplementary Fig. 2. Targeting capacity of naive OCs. (**a, b**) Schematic diagram of the targeting ability of naive OCs to soft tissue and ectopic calcification. OCs (2×10^4 per well) were seeded on one side of the well in a 48-well plate, and ectopic calcified tissue and soft tissues were separately seeded on the other side of the well. After 48 h, cells labelled with live/dead stain were observed under a microscope (10×). The results showed that only a very small number of naive cells could attach to calcified tissue, suggesting that naive OCs were poorly attracted to the calcified tissue. Live/dead staining images of the ability of naive OCs to bind soft tissues. The ability of naive OCs to bind soft tissues and ectopic calcified tissue was tested in parallel. Bar, 300 μm.

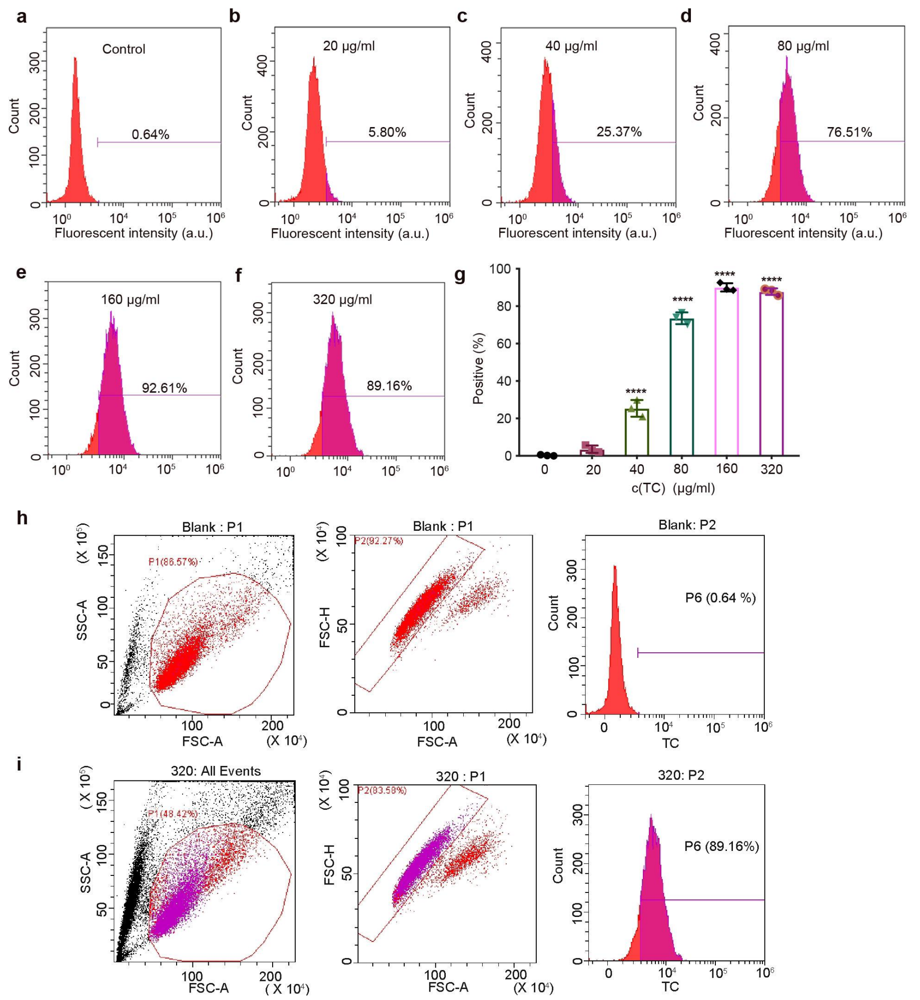

Supplementary Fig. 3. Analyses of TC-OCs engineering efficiency. (a-f) Representative flow cytometric analysis of TC-OCs treated with different concentrations of TC (0 μg/ml; 20 μg/ml; 40 μg/ml; 80 μg/ml; 160 μg/ml; 320 μg/ml). (**g**) Quantity of TC-positive cell after engineering with different concentrations of TC (n = 3 independent samples per group). Data are represented as mean ± SD. (****$p < 0.0001$) (**h, i**) The gating strategy of OCs and TC-OCs. Source data are provided as a Source Data file.

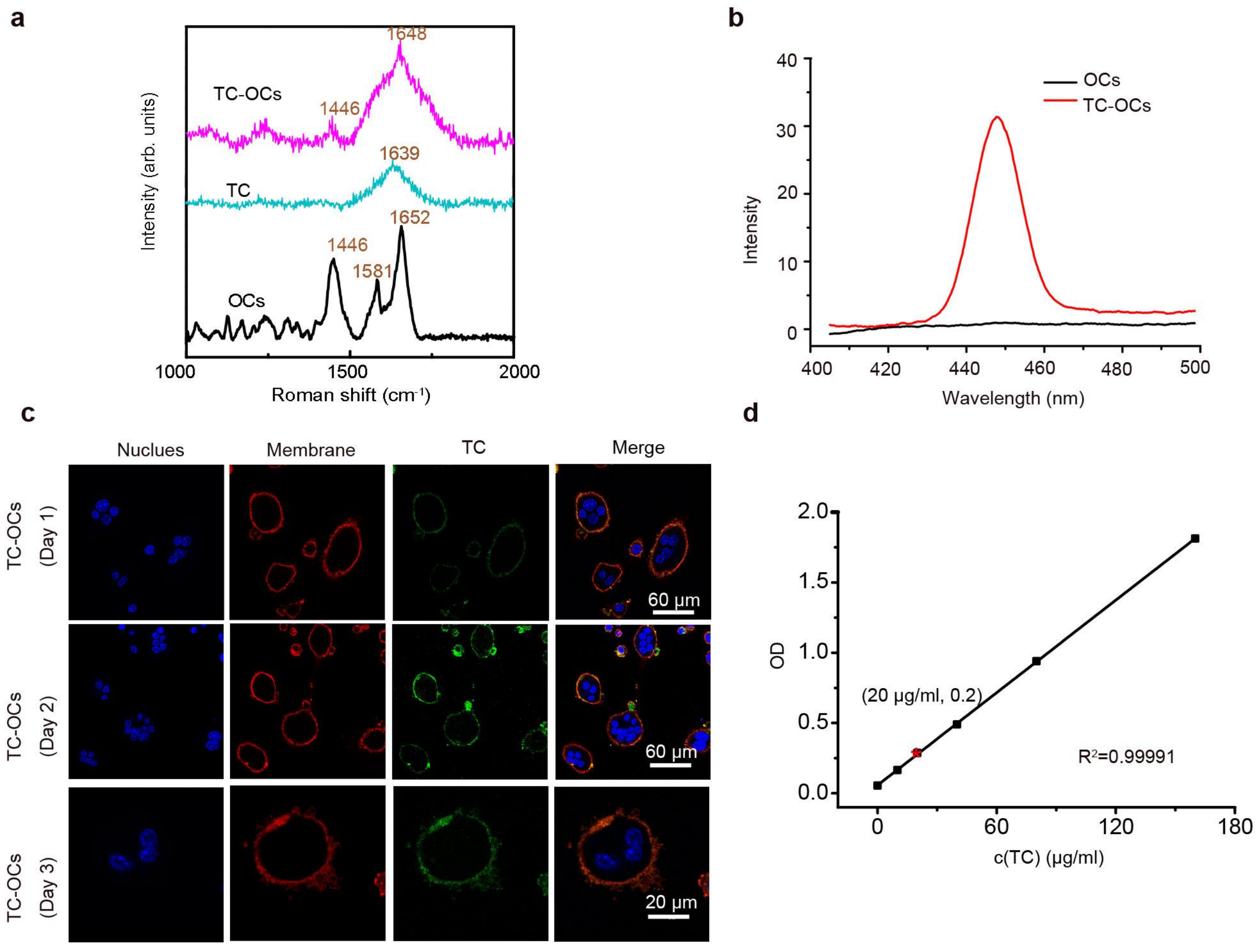

Supplementary Fig. 4. Analyses of engineered TC-OC characteristics and stability. (a) Roman shift analyses of TC for encapsulation of naive OCs. (**b**) Fluorescence intensity of OCs and TC-OCs as obtained on a fluorescence spectrophotometer. The results showed that TC was successfully engineered onto the cells. (**c**) Fluorescence detection of TC-OCs with CLSM on days 1, 2 and 3. (**d**) Linear curve obtained after modification. The red pentagram represented OD data for the TC-OC fluorescence intensity at the working concentration (160 μg/ml). Approximately 1.35×10^{11} TC molecules were bound to each OC.

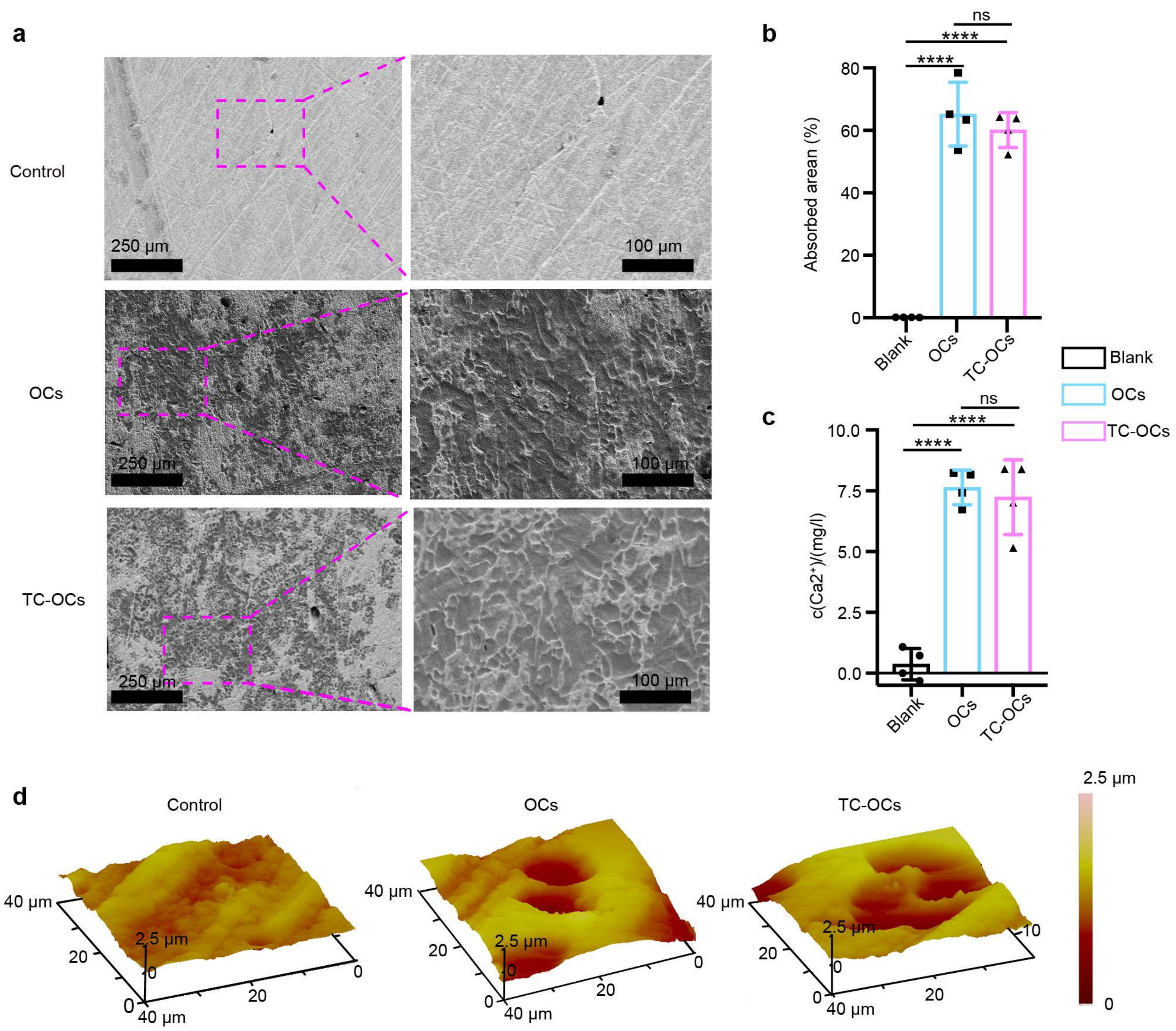

Supplementary Fig. 5. TC-Cs capacity verification. (**a**) These data also suggested that TC-engineered treatment had no negative influence on capacity of bone resorption according to the Pit Resorption Assay via culturing the OCs on the slice of bovine cortical bone; the results were confirmed by SEM (n = 4 independent samples per group). (**b**) Acid-etched area as measured by Image-J. Statistical analysis showed no distinguishable change in the resorption area between the OCs and TC-OCs groups (Blank vs OCs: ****$p < 0.0001$; Blank vs TC-OCs: ****$p < 0.0001$; OCs vs TC-OCs: ns $p = 0.5584$); (**c**) Ca^{2+} eluted from the acid-etched areas measured by ICP-OES also confirmed these results ($n = 4$ independent samples per group, Blank vs OCs: ****$p < 0.0001$; Blank

vs TC-OCs: ****$p < 0.0001$; OCs vs TC-OCs: $p = 0.8502$). (**d**) Examination of the resorption bone surface by AFM. Data are represented as mean ± SD, and analyzed by Ordinary one-way ANOVA. Source data are provided as a Source Data file. (****$p < 0.0001$)

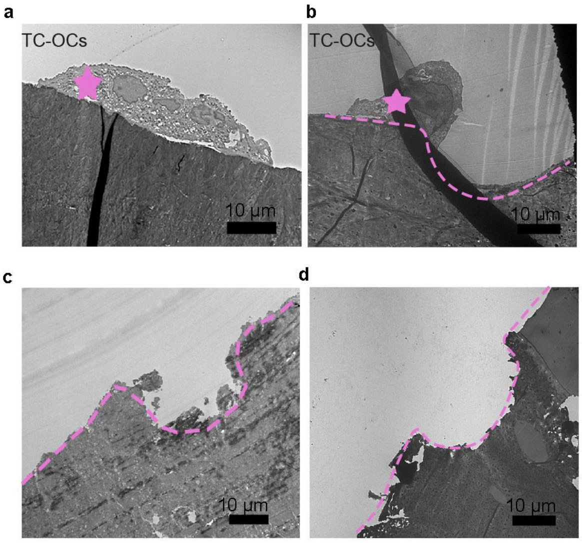

Supplementary Fig. 6. (**a**) TEM of bone resorption site treated with TC-OCs. TEM verified that the cells were multinuclear. (**b-d**) TEM images of bone resorption site of TC-OCs. According to observations from multiple fields of view, the depth of the pit ranges from 2.5-20 μm.

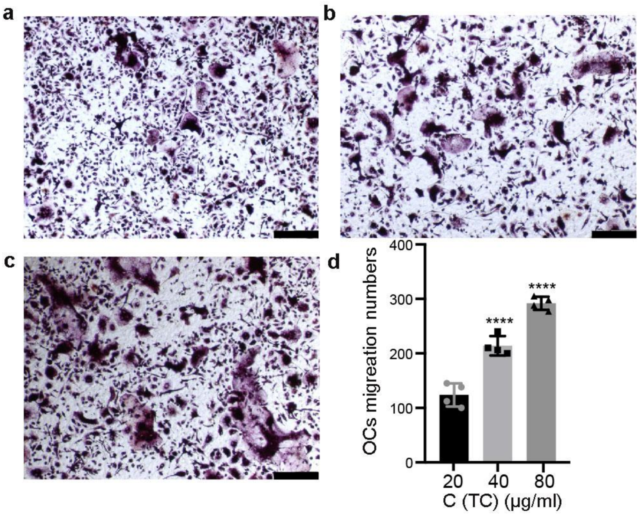

Supplementary Fig. 7. (**a-c**) Typical photography of Transwell-TRAP staining for TC-OCs encapsulated with different concentrations of 20 μg/ml, 40 μg/ml and 80 μg/ml. Scar bar: 200 μm (**d**)

Cell count in the field of view by Image J software ($n = 4$). Results have shown that with the increase of TC concentration, the cell migration ability is improved (Blank vs OCs: ****$p < 0.0001$; Blank vs TC-OCs: ****$p < 0.0001$). Data are represented as mean ± SD, and analyzed using Ordinary one-way ANOVA. Source data are provided as a Source Data file. (****$p < 0.0001$)

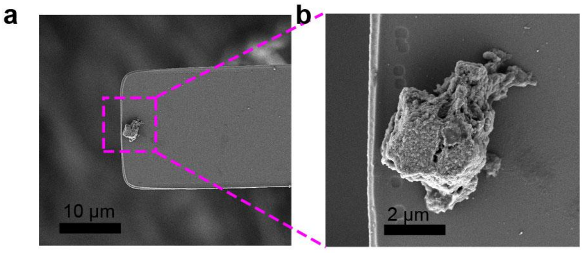

Supplementary Fig. 8. Calcified tissue powder was attached to a tipless cantilever observed by SEM.

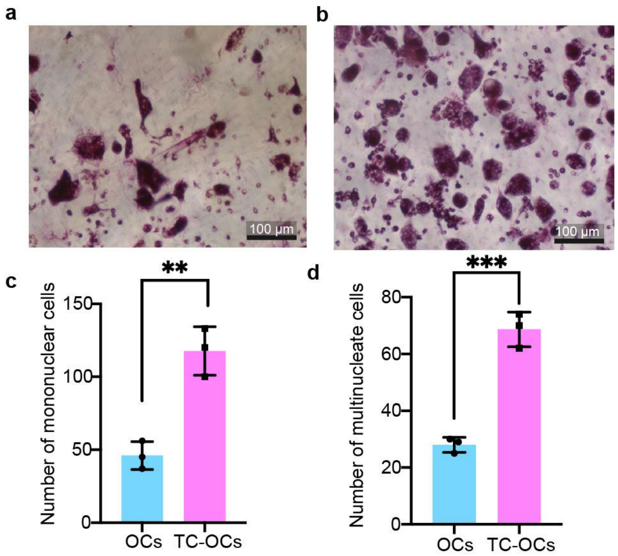

Supplementary Fig. 9. (**a, b**) TRAP staining images of OCs and TC-engineered OCs migrated to calcified tissue. Scale bar: 100 μm. (**c, d**) In OC/TC-OC groups, the number of probable mononuclear cells (OCs vs TC-OCs: **$p = 0.0029$) and multinucleated cells (OCs vs TC-OCs: ***$p = 0.0005$) that migrated to the calcified tissue in vitro ($n = 3$). Data are represented as mean ± SD, and analyzed using unpaired *t*-tests. Source data are provided as a Source Data file. (**$p < 0.01$, ***$p < 0.001$)

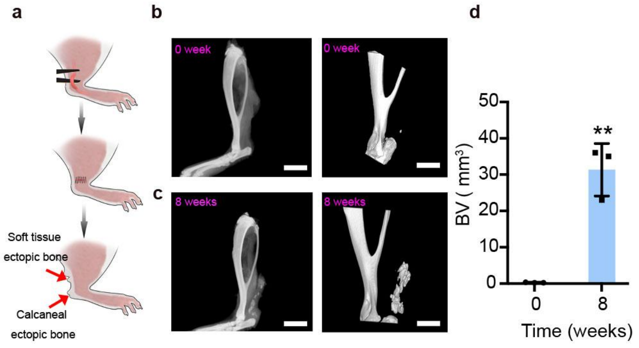

Supplementary Fig. 10. Analyses of Achilles tenotomy rats. (**a**) Trauma-induced model of HO in which mice received injury with hindlimb Achilles' tendon transection, resulting in HO formation. (**b-c**) Long bones in rats were examined by soft X-ray and three-dimensional Micro-CT after 8 weeks. (**d**) Quantitative analysis of BV on 0 and 8 weeks after injury (**p = 0.0017). Data were presented as mean ± SD, and analyzed using unpaired t-tests (n = 3 animals per group). Bar: 3 mm. Therefore, this animal model was successful and stable. Source data are provided as a Source Data file. (**p < 0.01)

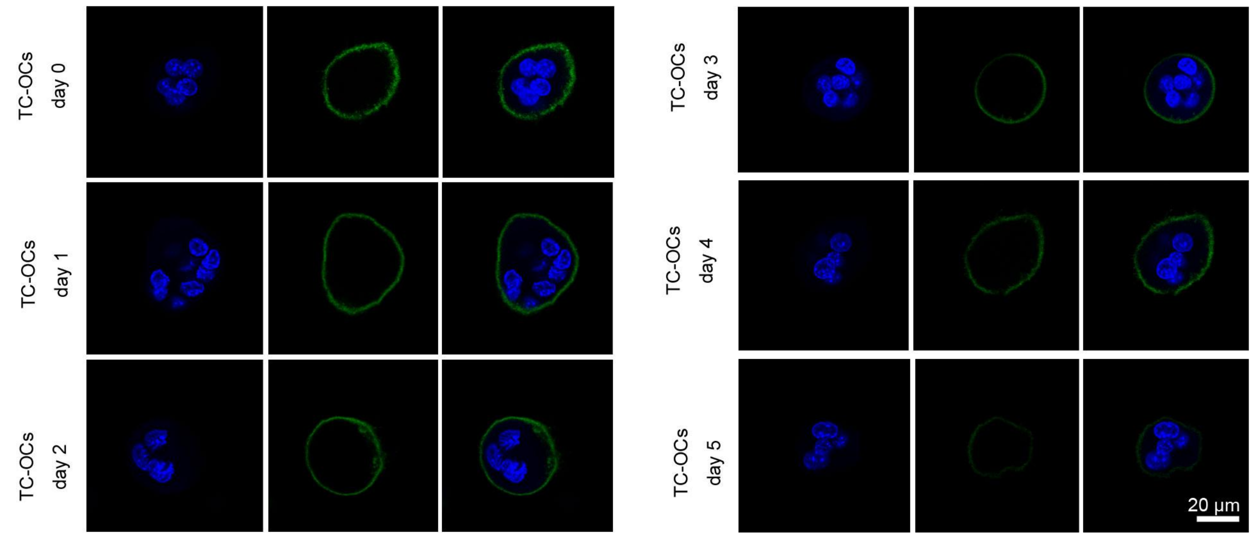

Supplementary Fig. 11. The stability of TC binding to osteoclasts in vivo. OCs engineered with TC (160 μg/ml) were injected into calcification site of tendon for four days. Then the cells were

washed by normal saline, and TC modified on the cell surface was monitored by CLSM at the following time points: 0 day, 1 days, 2 days, 3 days, 4 days and 5 days. The engineered OCs with TC cocoons on the cell surface could be stored stably in vivo over 4 days.

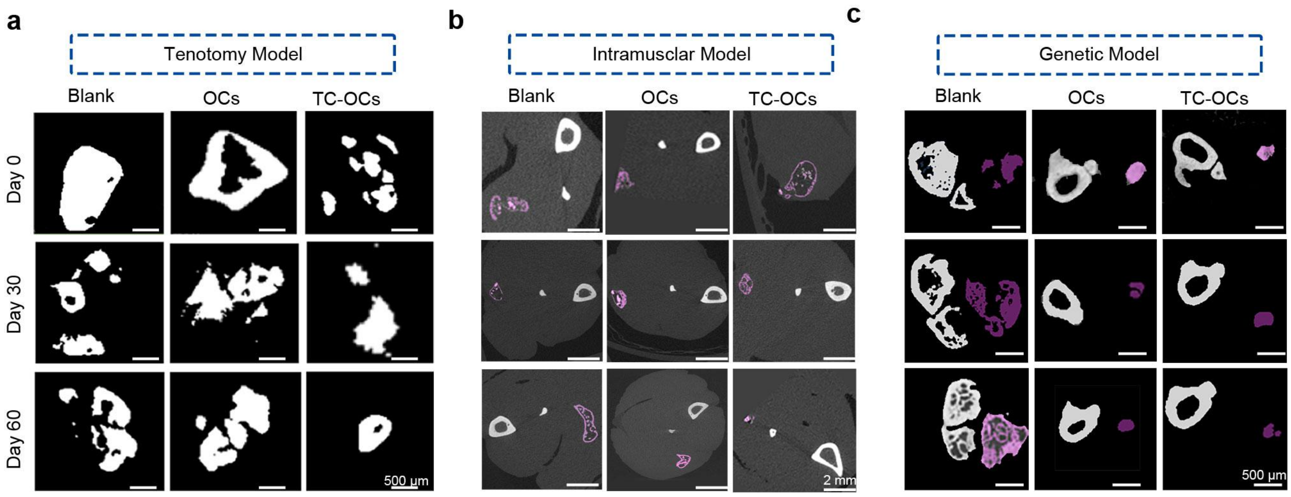

Supplementary Fig. 12. Two-dimensional micro-CT analyses of different types of HO. (**a**) Tenotomy model; The area indicated in the picture was the calcified site. (**b**) Intramuscular model; The read area indicated in the picture was the calcified site; (**c**) Genetic model; The read area indicated in the picture was the calcified site. In the three animal models, the calcification of the TC-OC group was the most significantly reduced.

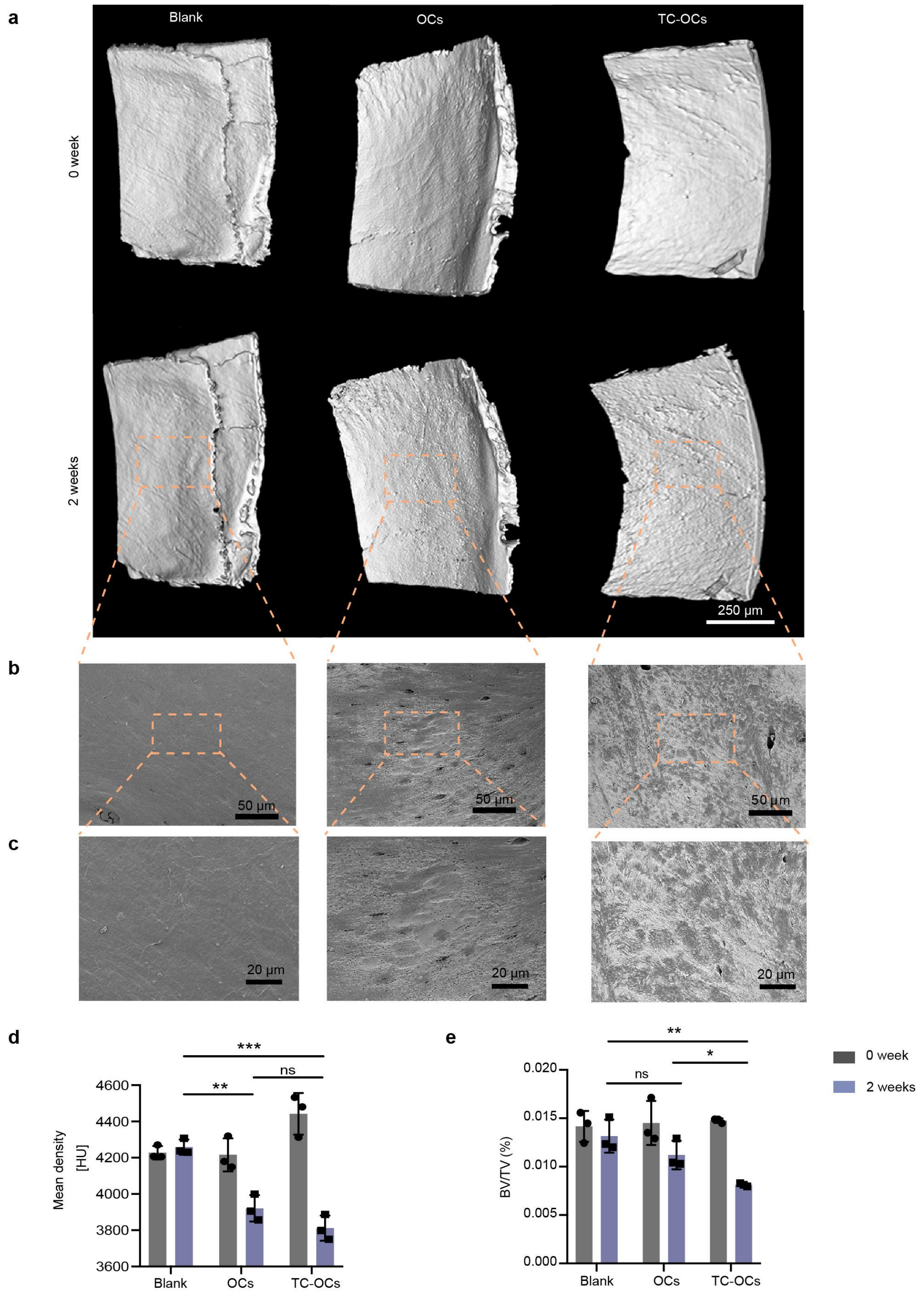

Supplementary Fig. 13. In vitro analysis of bone resorption in rat cranial bone as intramembranous bone. (**a**) 3D micro-CT reconstructed images of cranial bone treated with 0.9% NaCl, OCs, or TC-OCs at 2 weeks. (**b**) SEM images of cranial bone showed the increasing

resorption area and under different experimental conditions. (**c**) Enlarged views of a portion of (**b**). (**d, e**) Cranial bone parameters, including Mean density (MD, Hounsfield Units) and BV/TV were measured by micro-CT ($n = 3$ independent samples per group). Note that both MD and BV/TV of the TC-OC group were lower than those of the blank and OC groups.The MD value decreased from 4216.02 ± 91.19 units to 3920.79 ± 72.71 units in the OC groups, but the value decreased from 4442.20 ± 114.97 to 3811.81 ± 69.47 in the TC-OC groups; BMD after 2 weeks in Blank vs OCs: **p = 0.0014; Blank vs TC-OCs: ***p = 0.0003; OCs vs TC-OCs: ns p = 0.1656. The BV/TV value decreased from 0.015 ± 0.002 % to 0.011 ± 0.001 % in the OC groups but the BV/TV value decreased from 0.015 ± 2.34e-4 % to 0.008 ± 1.66e-4 % in TC-OC groups; BV/TV after 2 weeks in Blank vs. OCs: ns, $p = 0.2081$; blank vs. TC-OCs: **$p = 0.0071$; OCs vs. TC-OCs: *$p = 0.0227$. Data are represented as mean ± SD, and analysed by Ordinary one-way ANOVA. Source data are provided as a Source Data file. (*$p < 0.05$, **$p < 0.01$, ***$p < 0.001$)

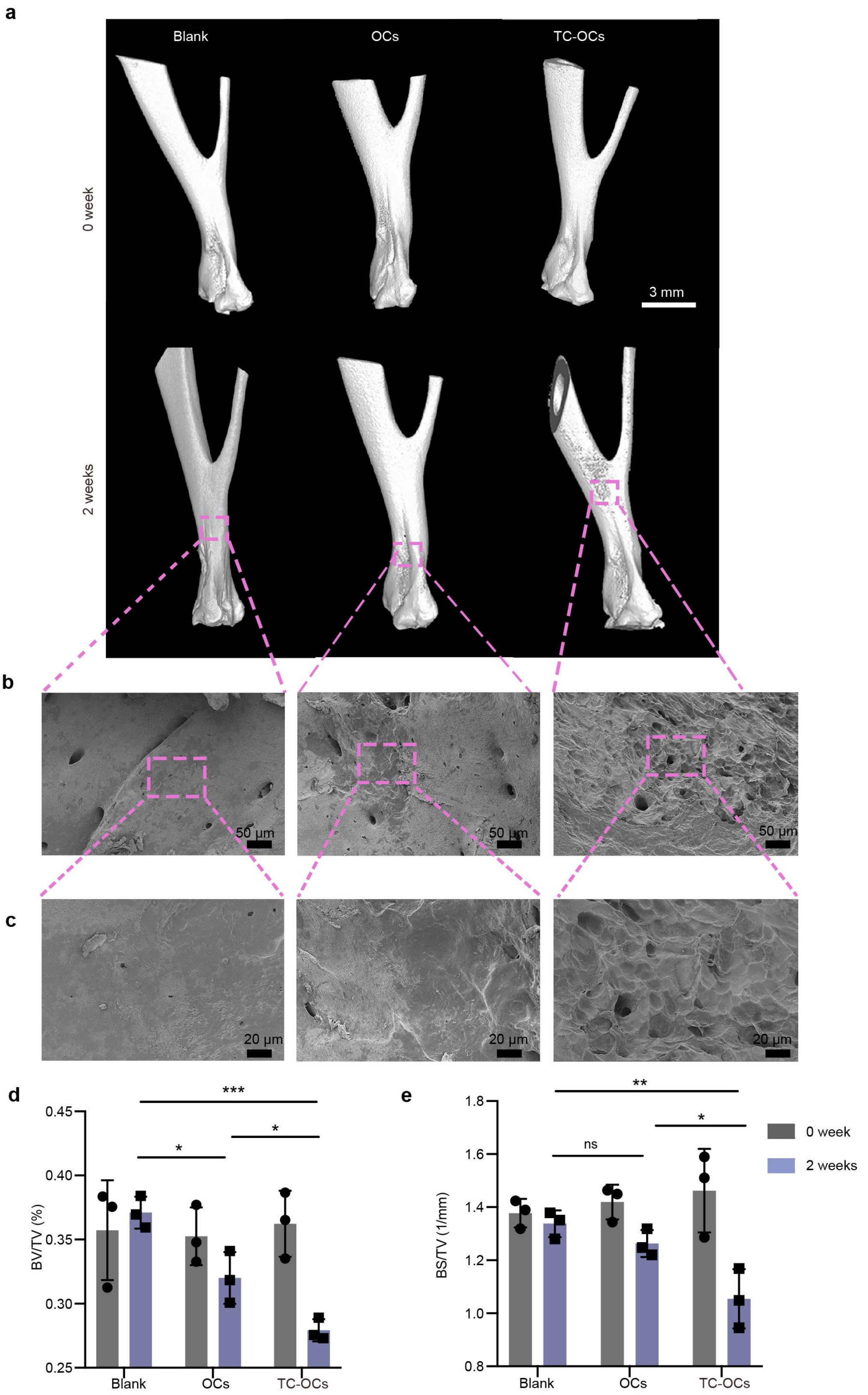

Supplementary Fig. 14. Qualitatively evaluate the efficiency of endochondral bone resorption by OCs and TC-engineered OCs. (a) 3D reconstruction views of tibial bone in the blank, OC, and

TC-OC groups after 2 weeks of treatment. (**b**) SEM images of tibia bone showed the increasing resorption area under different experimental conditions. (**c**) Enlarged images of (**b**). (**d, e**) Tibial bone parameters, including BV/TV and Bone Surface/Total Volume (BS/TV), were measured by micro-CT (n = 3 independent samples per group). The BV/TV and BS/TV values of the TC-OCs group were lower than those of the blank and OC groups, as expected. BV/TV value in the TC-OC groups decreased from 0.36 ± 0.03 % to 0.28 ± 0.01 %, but the BV/TV value in OCs group decreased from 0.35 ± 0.02 % to 0.32 ± 0.02 %; BV/TV after 2 weeks in Blank vs. OCs: *p = 0.0124; blank vs. TC-OCs: ***p = 0.0006; OCs vs. TC-OCs: *p = 0.0324. BS/TV was value in the TC-OC groups decreased from 1.46 ± 0.16 mm^{-1} to 1.05 ± 0.11 mm^{-1}, but the BS/TV value in OCs group decreased from 1.42 ± 0.07 mm^{-1} to 1.26 ± 0.05 mm^{-1}; BS/TV after 2 weeks in blank vs. OCs: ns, p=0.4982; blank vs. TC-OCs: **p=0.0095; OCs vs. TC-OCs: *p=0.0364. Data are represented as mean ± SD, and analysed by Ordinary one-way ANOVA. Source data are provided as a Source Data file. (*$p < 0.05$, **$p < 0.01$, ***$p < 0.001$)

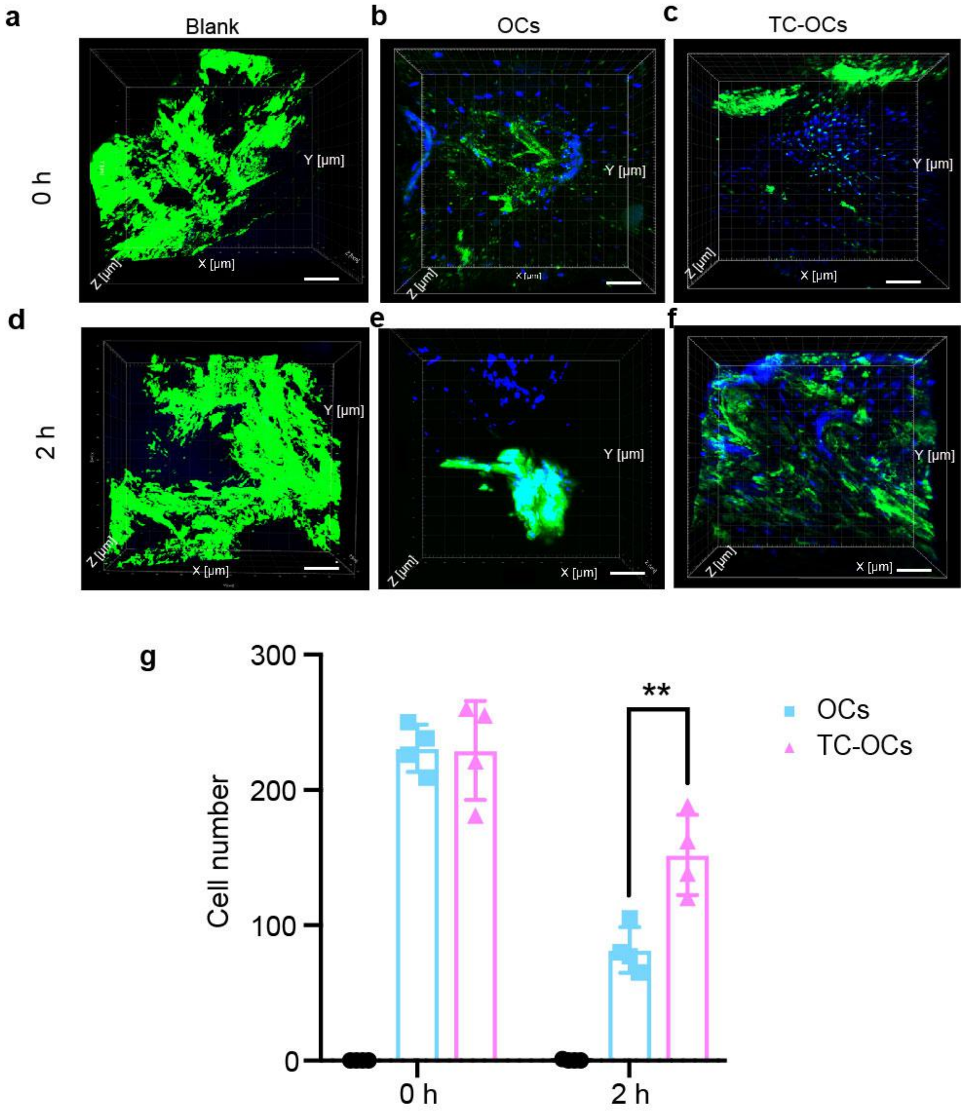

Supplementary Fig. 15. OCs/TC-OCs cell viability in vivo. (**a-f**) Two-photon images of ectopic calcified tissue at various imaging depths from rats injected with OCs or TC-OCs at 0 and 2 h. OCs (Hoechst 33258: blue) and calcification (calcein: green) (n = 4 samples per group, $^{**}p$=0.0061). Bar, 50 µm. (**g**) Quantitative analysis of migratory OCs/TC-OCs numbers. Data are represented as mean ± SD, and analyzed using unpaired t-tests. Source data are provided as a Source Data file. ($^{**}p$ < 0.01)

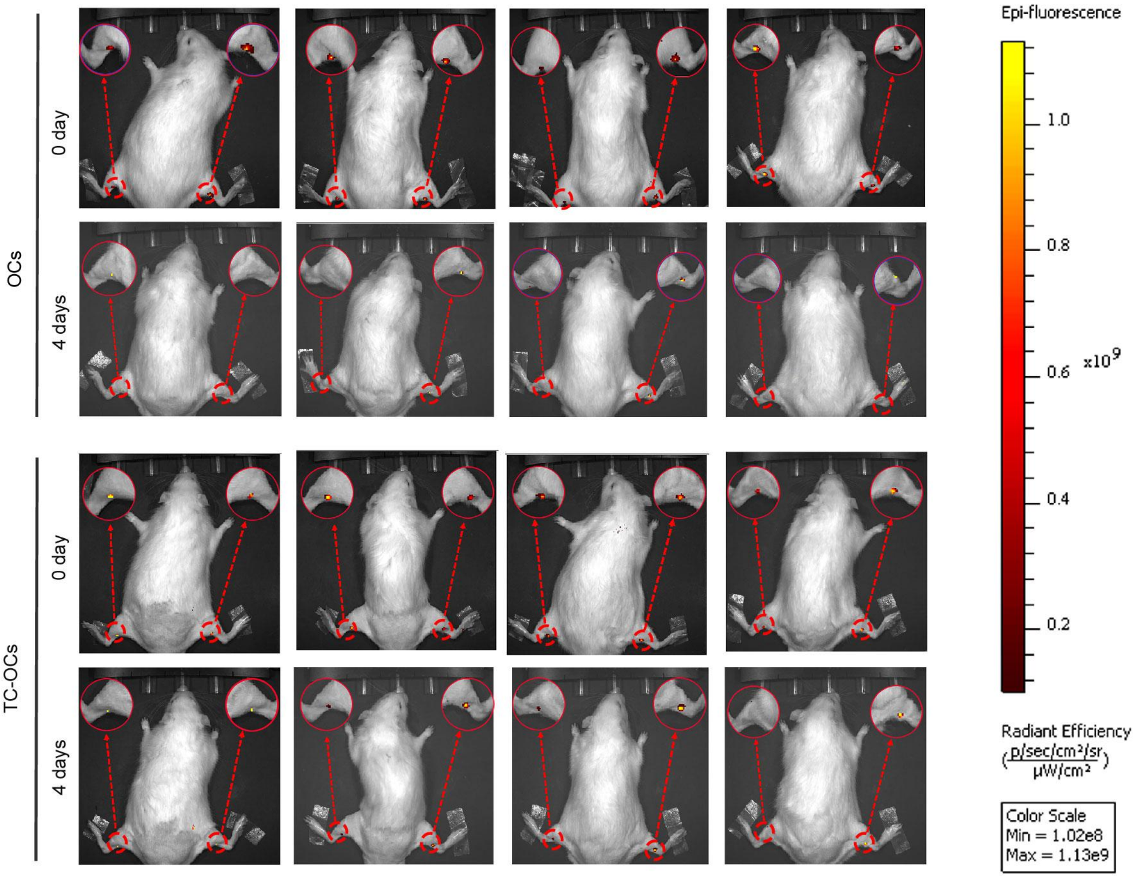

Supplementary Fig. 16. Qualitative evaluations of OCs and TC-engineered OCs viability in vivo. The florescence of OCs/TC-OCs (labelled with Cell Trace Far Red DDAO-SE fluorescent tag, a fixable, far-red-fluorescent tracer for long-term cell labeling) in rat tendon determined by in vivo (whole rats) and ex vivo imaging at 0 and 4 days. The intensity of florescence in the TC-OC group was stronger than that in the OC group after 4 days, implying that the cell viability of TC-OCs was superior to that of OCs (n = 4 rats per group).

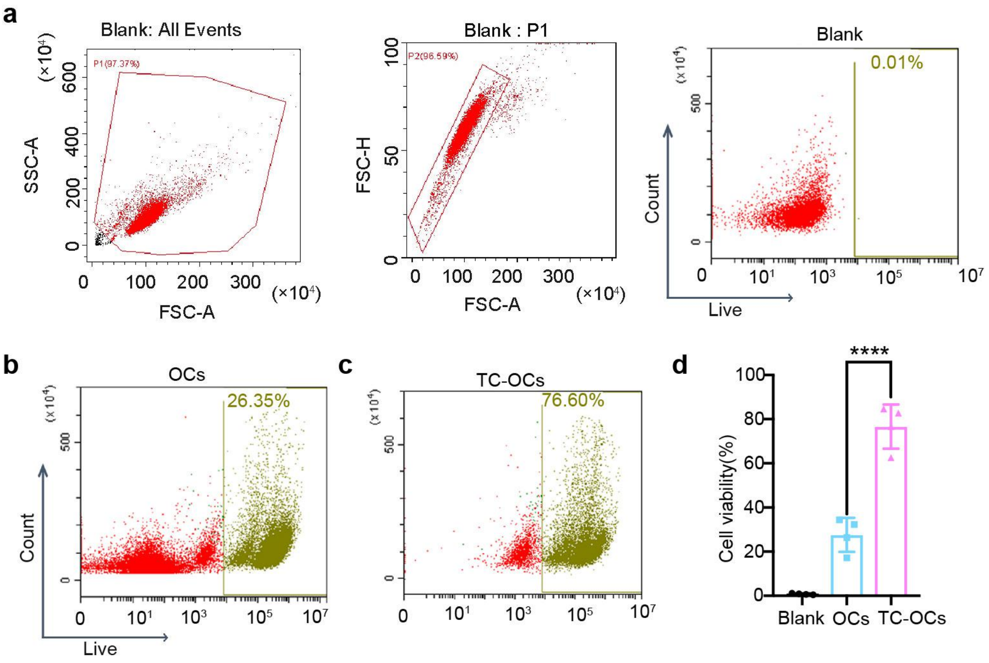

Supplementary Fig. 17. Quantitative evaluations of osteoclast and TC-engineered osteoclast cell viability in vivo. (**a-c**) Flow cytometry analysis of native OCs and surface-engineered OCs labeled with live/dead kits cell viability in vivo (n = 4 samples per group, ****$p < 0.0001$). (**d**) Quantitative analysis of OCs and TC-engineered OCs cell viability after four days. Data are represented as mean ± SD, and and analysed by Ordinary one-way ANOVA. Source data are provided as a Source Data file. (****$p < 0.0001$)

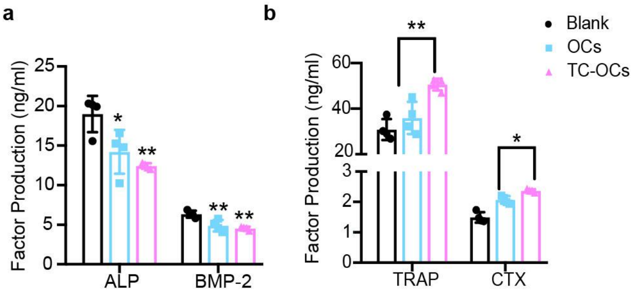

Supplementary Fig. 18. (**a, b**) Levels of ALP, BMP-2, TRAP and CTX were measured using ELISA kits (see Methods) in lysates derived from 4 individual tendons from intramuscular model rats treated with Blank, OCs and TC-OCs, respectively (n = 4 independent samples/group). Data are

represented as mean ± SD. ALP production, Blank vs OCs: $*p = 0.0247$; Blank vs TC-OCs: $**p = 0.0040$; BMP-2 production, Blank vs OCs: $**p = 0.0066$; Blank vs TC-OCs: $**p = 0.0017$; TRAP production, OCs vs TC-OCs: $**p = 0.0072$. CTX production, Blank vs OCs: OCs vs TC-OCs: $*p = 0.0192$. The statistical significance of data was determined by ordinary one-way ANOVA. Source data are provided as a Source Data file. ($*p < 0.05$, $**p < 0.01$)

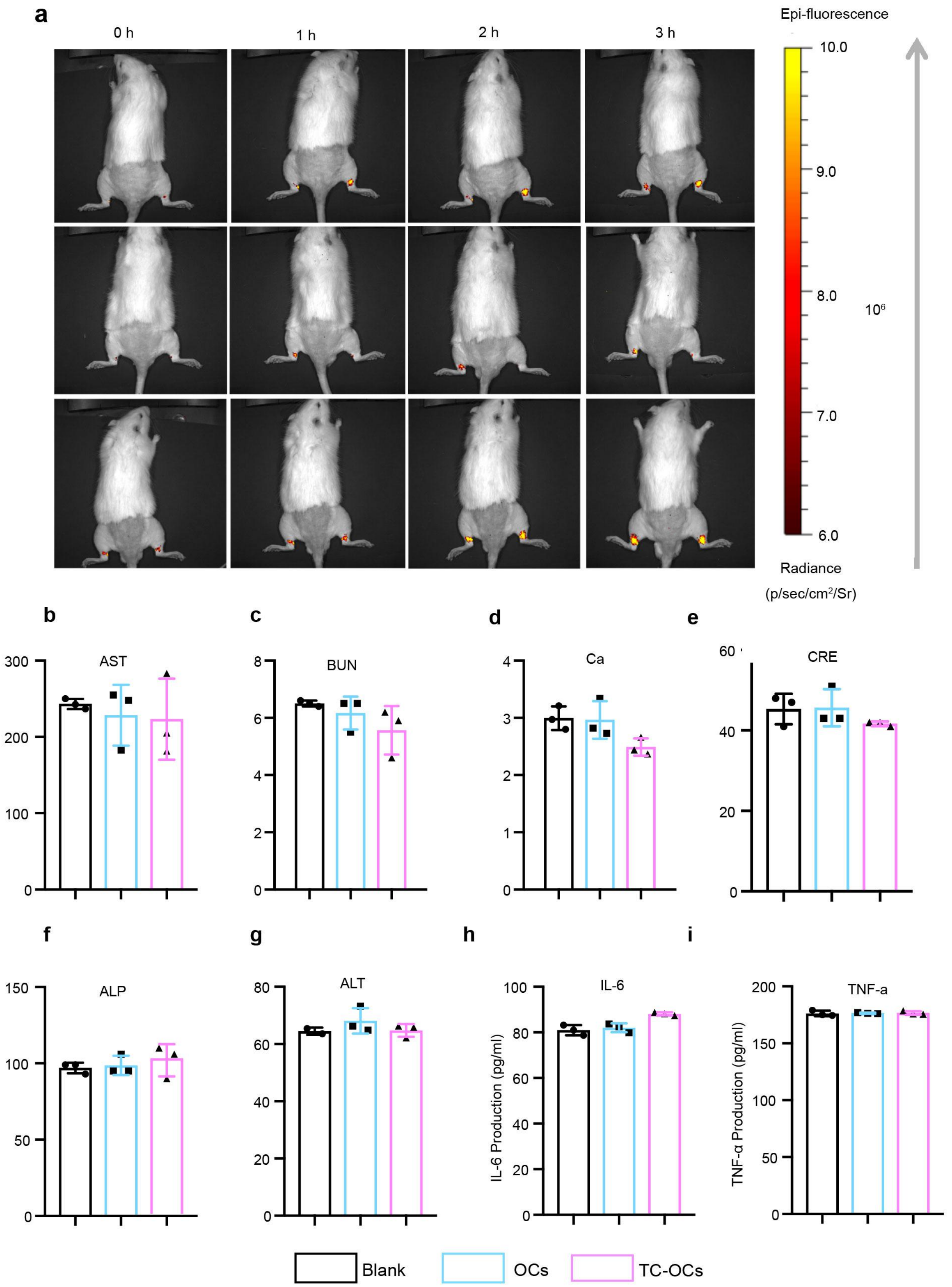
a
0 h
1 h
2 h
3 h
Epi-fluorescence
10.0
9.0
8.0
7.0
6.0
10^6
Radiance
(p/sec/cm²/Sr)
b
AST
c
BUN
d
Ca
e
CRE
f
ALP
g
ALT
h
IL-6
IL-6 Production (pg/ml)
i
TNF-a
TNF-α Production (pg/ml)
Blank
OCs
TC-OCs

Supplementary Fig. 19. Safety of the materials and biochemical analysis of serum. (**a**) Distribution of TC-OCs (labelled with 5 mM DiR, AAT Bioquest) in rats, as determined by in vivo (whole body) imaging every 1 h for a total of 3 h (*n* = 3 samples per groups). (**b-g**) Serum markers of OCs- and TC-OCs-treated rats were measured. The analyses showed that values of aspartate transaminase(AST), urea nitrogen (BUN), total calcium (Ca), and creatinine (CRE), as well as alkaline phosphatase (ALP) and alanine aminotransferase (ALT), did not change, which indicated the safety of TC-OCs (*n* = 3 samples per groups). (**h, i**) The levels of IL-6 and TNF-α in the sera of model rats were determined via ELISA kits (*n* = 3 samples per groups). Data are represented as mean ± SD. Source data are provided as a Source Data file.

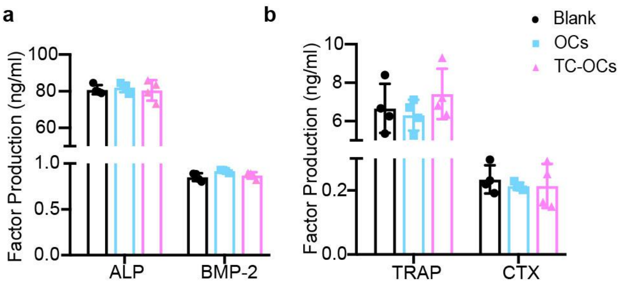

Supplementary Fig. 20. Protein-level analysis of bone formation and resorption after injection. (**a, b**) Levels of ALP, BMP-2, CTX and TRAP in the sera of model rats treated with 0.9% NaCl (blank), OCs and TC-OCs. Data are represented as mean ± SD (*n* = 4 independent samples/group). Source data are provided as a Source Data file.

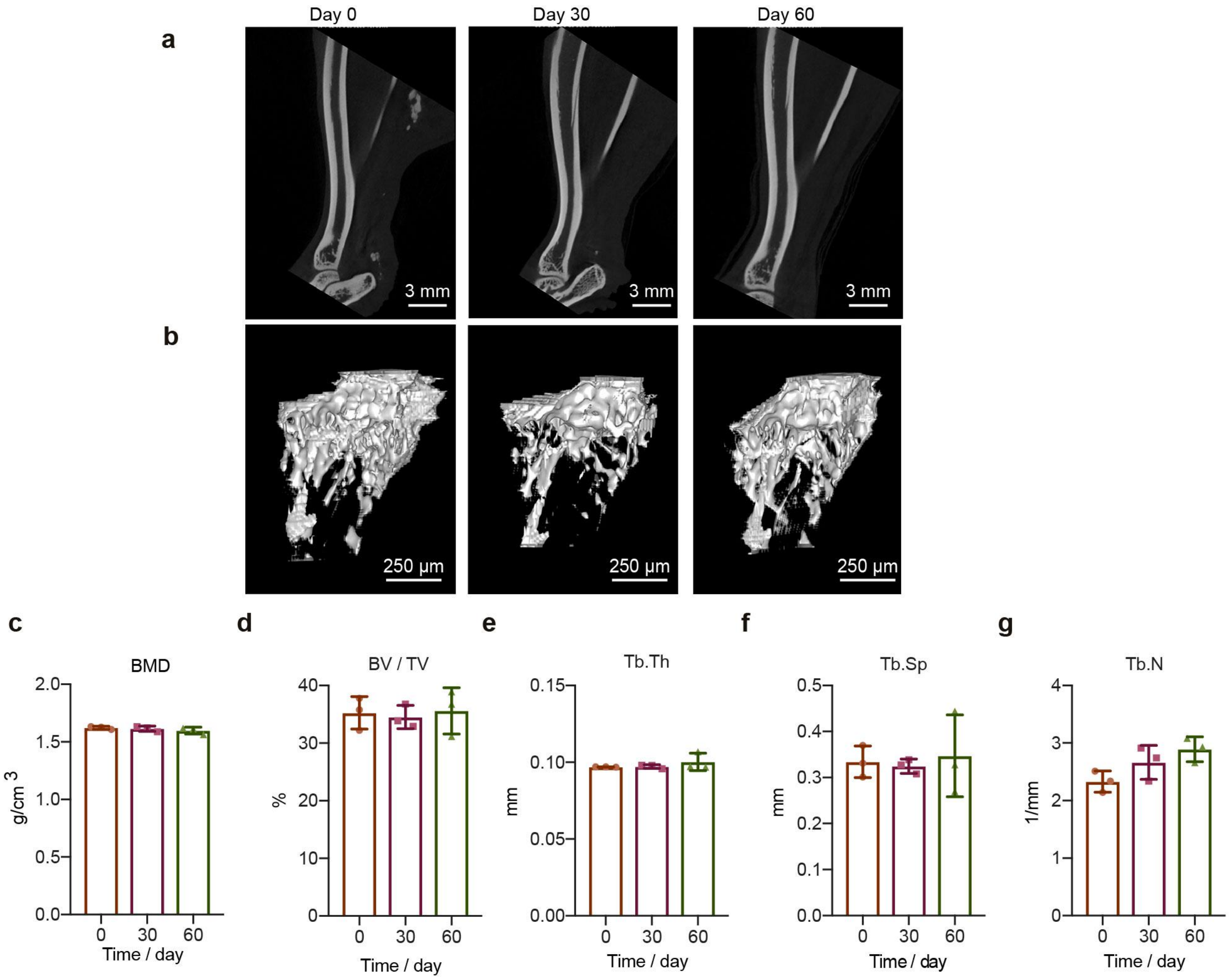

Supplementary Fig. 21. Analyses of normal bone structure and density in tenotomy model injected with TC-OCs. (**a**) Long bones in rats receiving TC-OCs were examined by soft X-ray. (**b**) 3D micro-CT. (**c-g**) Data were used to determine the BMD, BV /TV, Tb. Th, Tb. Sp and Tb. N. No major differences in these parameters were appreciable between rats treated with TC-OCs at different time, suggesting TC-OCs had no side effect on normal bone. Data are represented as mean ± SD (n = 3 rats/group). Source data are provided as a Source Data file.

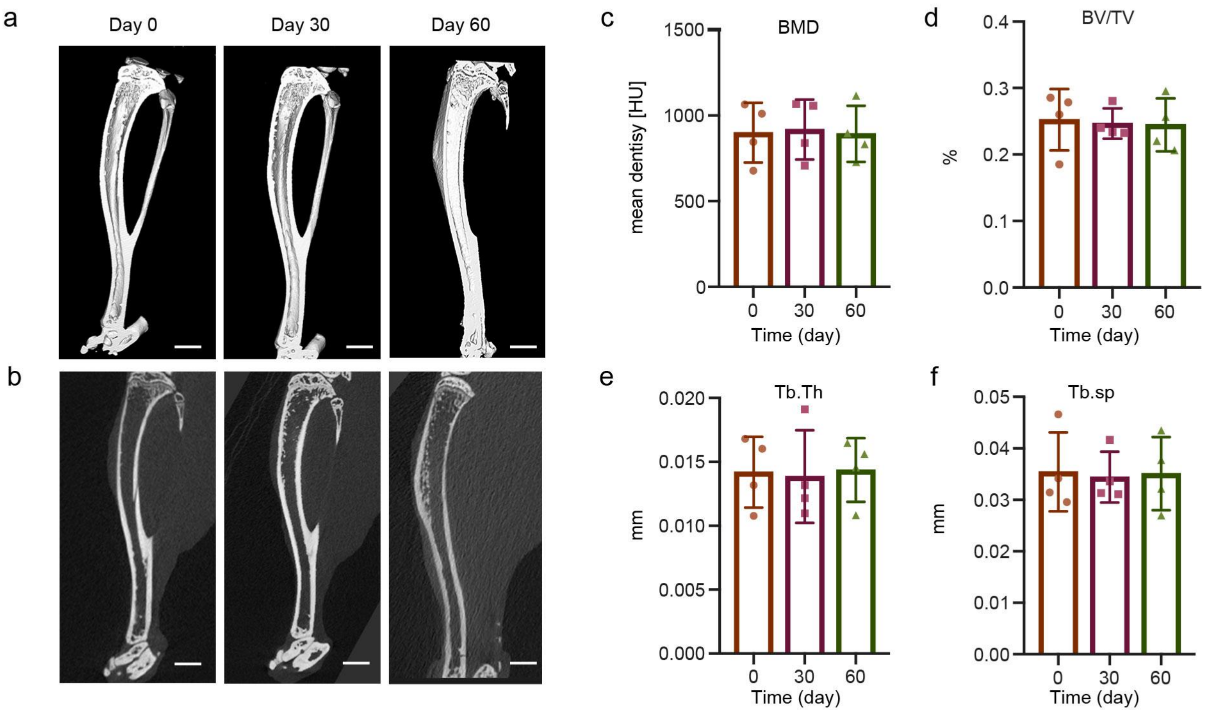

Supplementary Fig. 22. Analyses of normal bone structure and density in genetic model injected with TC-OCs. (**a**) Long bones in mice receiving TC-OCs were examined by micro-CT. Scale bar: 1 mm. (**b**) two-dimensional images of micro-CT. (**c-f**) Data were used to determine BMD, BV/TV, Tb. Th and Tb. Sp (n = 4 animals per groups). No major differences in these parameters were appreciable between rats treated with TC-OCs at different time, suggesting TC-OCs had no side effect on normal bone in genetic model. Data are represented as mean ± SD. Source data are provided as a Source Data file.

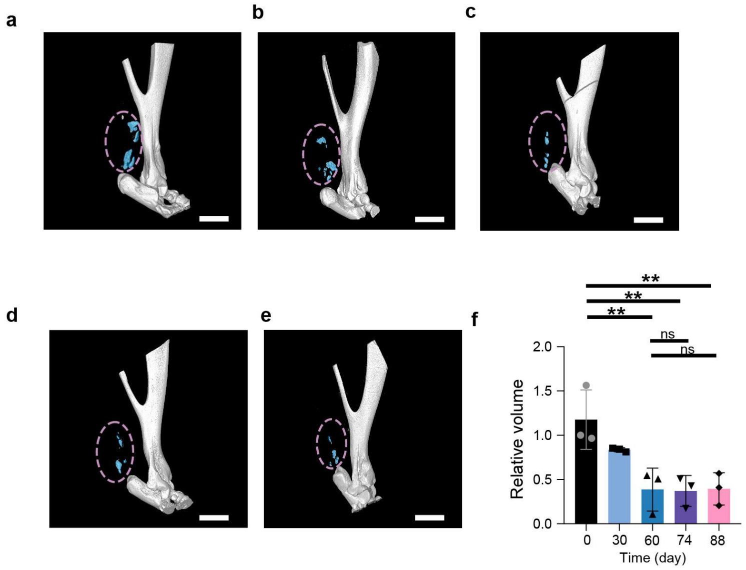

Supplementary Fig. 23. Analysis of rebound effects. (**a-c**) Rats were treated with TC-OCs day 0; day 30; day 60; Scale bar: 3 mm. (**d, e**) After the treatment termination, HO was evaluated by micro-CT at different time points 14 days, 28 days. Scale bar: 3 mm. (**f**) Quantity analysis of HO relative volume at different time points (n = 3 animals per groups). The results showed that there was no "rebound" effect after the treatment termination (day 0 vs. day 60: $**p = 0.0085$; day 0 vs. day 74 $**p = 0.0075$; day 0 vs. day 88: $**p = 0.0091$; day 60 vs day 74: ns, $p > 0.9999$; day 60 vs day 88: ns, $p > 0.9999$). Data are represented as mean ± SD. The statistical significance of data was determined by ordinary one-way ANOVA. Source data are provided as a Source Data file. ($**p < 0.01$)

References

1. Xu, J. C., *et al.* Leptin expression by heterotopic ossification-isolated tissue in rats with Achilles' tenotomy. *Saudi. Med. J,* **30**, 605-610 (2009).
2. Shimono, K., *et al.* Potent inhibition of heterotopic ossification by nuclear retinoic acid receptor-γ agonists. *Nat. Med.* **17**, 454 (2011).
3. Zhu, X., *et al.* Ultrafast optical clearing method for three-dimensional imaging with cellular resolution. *Proc. Natl. Acad. Sci., India* **116**, 11480-11489 (2019).
4. Khoury, D. S., *et al.* Host-mediated impairment of parasite maturation during blood-stage Plasmodium infection. *Proc. Natl. Acad. Sci., India* **114**, 7701-7706 (2017).
