# Supplementary material for: Engineered osteoclasts as living treatment materials for heterotopic ossification therapy
Source: Nat Commun. 2021 Nov 3;12:6327. doi: 10.1038/s41467-021-26593-1 (PMC8566554; doi:10.1038/s41467-021-26593-1)
Supplement: Supplementary file 3 — Description of Additional Supplementary Files [file 41467_2021_26593_MOESM3_ESM.pdf]

Description of Additional Supplementary Movie

**Supplementary** Movie 1. The cells in the OC group could not adhere firmly to the tipless cantilever as it moved within calcified tissue.

**Supplementary** Movie 2. The cells in the TC-OC group attached tightly to the tipless cantilever as it moved within calcified tissue.
