# Supplementary material for: Engineered osteoclasts as living treatment materials for heterotopic ossification therapy
Source: Nat Commun. 2021 Nov 3;12:6327. doi: 10.1038/s41467-021-26593-1 (PMC8566554; doi:10.1038/s41467-021-26593-1)
Supplement: Supplementary file 6 — Reporting summary [file 41467_2021_26593_MOESM6_ESM.pdf]

nature portfolio

Corresponding author(s): Zhijian Xie and Ruikang Tang

Last updated by author(s): Oct 1, 2021

Reporting Summary

Nature Portfolio wishes to improve the reproducibility of the work that we publish. This form provides structure for consistency and transparency in reporting. For further information on Nature Portfolio policies, see our Editorial Policies and the Editorial Policy Checklist.

Statistics

For all statistical analyses, confirm that the following items are present in the figure legend, table legend, main text, or Methods section.

n/a	Confirmed	
☐	☒	The exact sample size (n) for each experimental group/condition, given as a discrete number and unit of measurement
☐	☒	A statement on whether measurements were taken from distinct samples or whether the same sample was measured repeatedly
☐	☒	The statistical test(s) used AND whether they are one- or two-sided<br>*Only common tests should be described solely by name; describe more complex techniques in the Methods section.*
☒	☐	A description of all covariates tested
☐	☒	A description of any assumptions or corrections, such as tests of normality and adjustment for multiple comparisons
☐	☒	A full description of the statistical parameters including central tendency (e.g. means) or other basic estimates (e.g. regression coefficient) AND variation (e.g. standard deviation) or associated estimates of uncertainty (e.g. confidence intervals)
☐	☒	For null hypothesis testing, the test statistic (e.g. F, t, r) with confidence intervals, effect sizes, degrees of freedom and P value noted<br>*Give P values as exact values whenever suitable.*
☒	☐	For Bayesian analysis, information on the choice of priors and Markov chain Monte Carlo settings
☐	☒	For hierarchical and complex designs, identification of the appropriate level for tests and full reporting of outcomes
☒	☐	Estimates of effect sizes (e.g. Cohen's d, Pearson's r), indicating how they were calculated

Our web collection on statistics for biologists contains articles on many of the points above.

Software and code

Policy information about availability of computer code

Data collection	Nanoscope IVa, Veeco 6.12r1 was used to collect AFM data.<br>HO of animals model were subjected to X-ray analysis (Faxitron MX-20, USA), micro-CT (Suzhou Hiscan Information Technology Co., Ltd.) and u-CT (MiLabs, Netherlands).<br>CLSM (FV1000, Olympus) was used to observe TC molecule.<br>CytoFLEX LX, Beckman Coulter, US<br>scanning electron microscope (SEM) (SU-8010 Hitachi Co, Tokyo, Japan)<br>FTIR spectra(Shimadzu, Japan)<br>Fluorescence spectrophotometry (RF-5301, Shimadzu, Tokyo, Japan)
Data analysis	Image J (version 1.6.0)was used to analyze the fluorescence images and bone resorption area.<br>FlowJo vX.0.7 was used for flow data analysis.<br>The 3D CLSM images were obtained by Imaris 9.5(Batch) software.<br>3D images of HO were reconstructed with Hiscan Reconstruct software (version 1.0, Suzhou Hiscan Information Technology Co., Ltd.).<br>The BV of HO was assessed by Hiscan Analyser software (version 1.0, Suzhou Hiscan Information Technology Co., Ltd.) and IMALYTICS preclinical (Version 2.1.8.9).<br>The CLSM images were obtained by Olympus FluoView FV1000 version 2.1b<br>GraphPad Prism 8 data analysis shown in graphs.

For manuscripts utilizing custom algorithms or software that are central to the research but not yet described in published literature, software must be made available to editors and reviewers. We strongly encourage code deposition in a community repository (e.g. GitHub). See the Nature Portfolio guidelines for submitting code & software for further information.

Data

Policy information about availability of data

All manuscripts must include a data availability statement. This statement should provide the following information, where applicable:

- Accession codes, unique identifiers, or web links for publicly available datasets
- A description of any restrictions on data availability
- For clinical datasets or third party data, please ensure that the statement adheres to our policy

All data generated in this study are provided in the Supplementary Information/Source Data files. Source Data files have been deposited at zenodo database with open access (https://zenodo.org/record/5544088#.YVcldUZBxdg).

Field-specific reporting

Please select the one below that is the best fit for your research. If you are not sure, read the appropriate sections before making your selection.

[x] Life sciences [ ] Behavioural & social sciences [ ] Ecological, evolutionary & environmental sciences

For a reference copy of the document with all sections, see nature.com/documents/nr-reporting-summary-flat.pdf

Life sciences study design

All studies must disclose on these points even when the disclosure is negative.

Sample size	Samples sizes were determined based on previous experience and by referencing previously published studies.
Data exclusions	No data were excluded from the analyses.
Replication	All experiments described in this paper have been done more than triplicates. All the replication attempts were successful.
Randomization	Animals were randomly assigned to receive osteoclasts or TC engineered osteoclasts. For in vitro ectopic calcification resorption studies, samples were allocated in different groups depending on the volume of ectopic calcification.
Blinding	Investigators were blinded to group allocation during data collection of osteoclasts counts and analysis. Quantification of the resorption area of OCs and TC-OCs group was performed blinded. In vivo experiments, the identity (treatment condition) of each animal was blinded when measurements were collected (the investigators were blinded to group allocation during data collection and analysis).

Reporting for specific materials, systems and methods

We require information from authors about some types of materials, experimental systems and methods used in many studies. Here, indicate whether each material, system or method listed is relevant to your study. If you are not sure if a list item applies to your research, read the appropriate section before selecting a response.

Materials & experimental systems

n/a	Involved in the study	
[x]	[ ]	Antibodies
[x]	[ ]	Eukaryotic cell lines
[x]	[ ]	Palaeontology and archaeology
[ ]	[x]	Animals and other organisms
[x]	[ ]	Human research participants
[x]	[ ]	Clinical data
[x]	[ ]	Dual use research of concern

Methods

n/a	Involved in the study	
[x]	[ ]	ChIP-seq
[ ]	[x]	Flow cytometry
[x]	[ ]	MRI-based neuroimaging

Animals and other organisms

Policy information about studies involving animals; ARRIVE guidelines recommended for reporting animal research

Laboratory animals	Six to eight-week-old male mice C57BL/6 and eight-week-old male Sprague–Dawley rats (body weight 290–330 g) and six -week-old male Sprague–Dawley rats provided by Department of Orthopaedic Surgery, Sir Run Run Shaw Hospital, Zhejiang University School of Medicine. Eight-week-old male the Mkx-/- transgenic mice were provided by Dr. Ronen Schweitzer (Oregon 20 Health and Science University, Portland, OR). All animals were housed under a 12 h light/dark cycle at controlled room temperature of 22-24°C and a relative humidity of 40-70 %.
Wild animals	The study did not involve wild animals.

Field-collected samples	The study did not involve samples collected from the field
Ethics oversight	All animal care and handing procedures were in strict accordance with the guidelines approved by the Institutional Animal Ethics Committee of Sir Run Run Shaw Hospital of Zhejiang University (Number: SRRSH2021401 and 201801224).

Note that full information on the approval of the study protocol must also be provided in the manuscript.

Flow Cytometry

Plots

Confirm that:

- [x] The axis labels state the marker and fluorochrome used (e.g. CD4-FITC).
- [x] The axis scales are clearly visible. Include numbers along axes only for bottom left plot of group (a 'group' is an analysis of identical markers).
- [x] All plots are contour plots with outliers or pseudocolor plots.
- [x] A numerical value for number of cells or percentage (with statistics) is provided.

Methodology

Sample preparation	Mature osteoclasts were differentiated from bone marrow-derived macrophages (BMMs) derived from C57 mice tibiae and femurs. Control and TC-engineered cells were stained with 250 nM PKH26 for general cell membrane labelling for 3 min and with 5 µM Hoechst 33258 for another 3 min, with no TC treatment. The stained cells were washed, collected, and adjusted to 300 µl of cell suspension solution (1×106 cell/ml), and then analysed on an CytoFLEX LX.
Instrument	CytoFLEX LX, Beckman Coulter, US
Software	(FlowJo vX.0.7).
Cell population abundance	No cell sorting was performed.
Gating strategy	10,000 cells were analyzed in each condition. The gating was first performed using a FCS/SSC dot plot. The gain used was fixed when all populations was observed on the plot. The main population was gated to perform a singlets plot using FSC-H and FSC-A parameters.

- [x] Tick this box to confirm that a figure exemplifying the gating strategy is provided in the Supplementary Information.
